# Solitary Fibrous Tumors of the Head and Neck: A Report of Two Cases and Review of the Literature

**DOI:** 10.7759/cureus.101396

**Published:** 2026-01-12

**Authors:** Jafar Hayat, Altaf A Alfadhly, Yasmeen Alshahoumi, Smiley-Annie George, Fareed Al Qusous, Maha Al-Gilani

**Affiliations:** 1 Surgery, Ministry of Health, Kuwait City, KWT; 2 College of Medicine, Kuwait University, Kuwait City, KWT; 3 General Surgery, Jaber Al-Ahmad Hospital, Zahra, KWT; 4 Otolaryngology - Head and Neck Surgery, Jaber Al-Ahmad Hospital, Zahra, KWT; 5 General Practice, The Great Western Hospital, Bristol, GBR

**Keywords:** case series, cheek, floor of the mouth, head and neck neoplasms, solitary fibrous tumor

## Abstract

Solitary fibrous tumors (SFTs) are rare mesenchymal neoplasms, particularly uncommon in the cheek and floor of the mouth. We report two such cases to emphasize diagnostic and management considerations. A retrospective review at a tertiary academic hospital included two female patients aged 40 and 49 years. Both underwent clinical evaluation, radiologic imaging, surgical excision, and histopathologic assessment with immunohistochemistry, including STAT6, CD34, BCL2, and CD99. Risk stratification was based on mitotic index and histological features. The first case involved a 40-year-old woman with a 3.5 × 2 × 1.5 cm lesion in the floor of the mouth. Histopathology confirmed SFT with STAT6, CD34, BCL2, and CD99 positivity; the mitotic rate was 1/10 HPF, consistent with low risk. The second case involved a 49-year-old woman with a 1.5 × 1.5 cm right cheek mass, also confirmed as SFT with a similar immunoprofile and negligible mitotic activity, indicating very low risk. Both patients underwent complete excision and recovered uneventfully, with no recurrence on a two-week follow-up. These cases illustrate that SFTs, though rare in the cheek and floor of the mouth, should be considered in the differential diagnosis of soft tissue tumors. Diagnosis depends on histopathology and immunohistochemistry, particularly STAT6. Complete excision is usually curative, but long-term surveillance remains essential.

## Introduction

Solitary fibrous tumors (SFTs) are mesenchymal neoplasms characterized by fibroblastic differentiation [1]. First described in the pleura, they are now reported at nearly every anatomic site, including the head and neck region (HNR), where head and neck SFTs (HNSFTs) comprise about 6%-18% of all cases [2]. Within the HNR, they occur most often in the sinonasal tract, orbit, oral cavity, and neck [3]. Diagnosis is particularly challenging when they arise in less common oral locations, such as the buccal mucosa, cheek, or floor of the mouth.

SFTs are usually asymptomatic and slow growing but may cause localized swelling or discomfort, and rarely symptoms such as pain or facial paralysis depending on location [2]. Histopathology typically demonstrates patternless cellularity with staghorn-shaped vessels, while immunohistochemistry (IHC) is essential, with CD34 and STAT6 serving as key markers [1].

Although traditionally viewed as benign, HNSFTs may behave aggressively, with potential for recurrence or metastasis, especially when large or incompletely excised. Recurrence has been reported in up to 40% of cases, with distant spread in a small subset [3]. This report describes two rare SFTs of the cheek and floor of the mouth, highlighting diagnostic features and management considerations.

## Case presentation

This case report is based on anonymized data collected from two individuals. All identifying information has been removed to ensure participant confidentiality and privacy. The data used in this study were gathered retrospectively, and no direct patient intervention occurred for this research. Written informed consent was obtained from both patients prior to the inclusion of their data in this case report. The patients were informed of the purpose of the study, the use of their anonymized data, and their rights to confidentiality and privacy.

Case 1

A 40-year-old woman presented with a lesion on the floor of the mouth, measuring 3.5 × 2 × 1.5 cm, which was electively excised under sterile conditions. A mouth prop was used to facilitate intraoral access. The lesion was identified and removed en bloc via an elliptical incision with clear macroscopic margins. Hemostasis was achieved, and closure was performed using 3-0 absorbable Vicryl sutures in a combination of interrupted and horizontal mattress patterns. The postoperative course was unremarkable. She was administered IV DNS at 100 mL/h for the first postoperative day, oral amoxicillin-clavulanate (1 g twice daily for 7 days), and mild analgesia. A soft diet was initiated on postoperative day one, with standard oral hygiene instructions.

Histopathological evaluation revealed a solitary fibrous tumor. Microscopic examination showed a moderately cellular, circumscribed spindle cell neoplasm composed of ovoid to fusiform cells within a fibrocollagenous stroma. Prominent staghorn-like vessels were identified along with scattered lymphocytes and mast cells. There was no evidence of atypia, necrosis, or mitotic activity exceeding 1/10 HPF.

**Figure 1:**
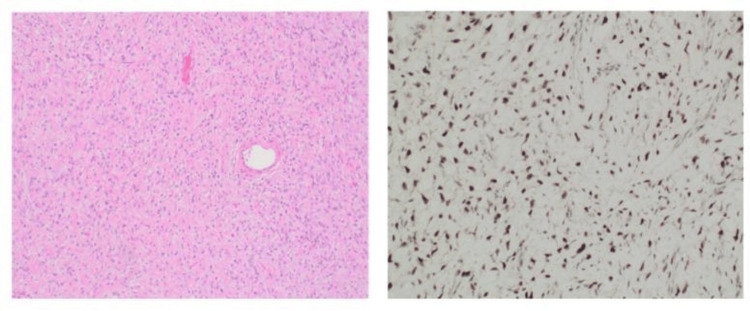
Moderate cellular spindle cell tumor with a fibrocollagenous stroma (H&E, ×400) and a STAT6 IHC stain showing nuclear positivity in the tumor cells.

Immunohistochemistry revealed tumor cells positive for CD34, BCL-2, CD99, and STAT6, and negative for SMA, Desmin, and S100, confirming the diagnosis. Margins were involved (tumor extended to the inked margin), but there was no evidence of lymphatic or vascular invasion, and no regional lymph nodes were submitted (Figure 1). Based on the established criteria [4], she was assigned a low-risk score (1 point).

During her two-week postoperative follow-up, she reported no pain, no changes in speech or taste, and normal tongue and jaw mobility. The surgical site appeared well-healed, with no signs of infection or recurrence.

Case 2

**Figure 2:**
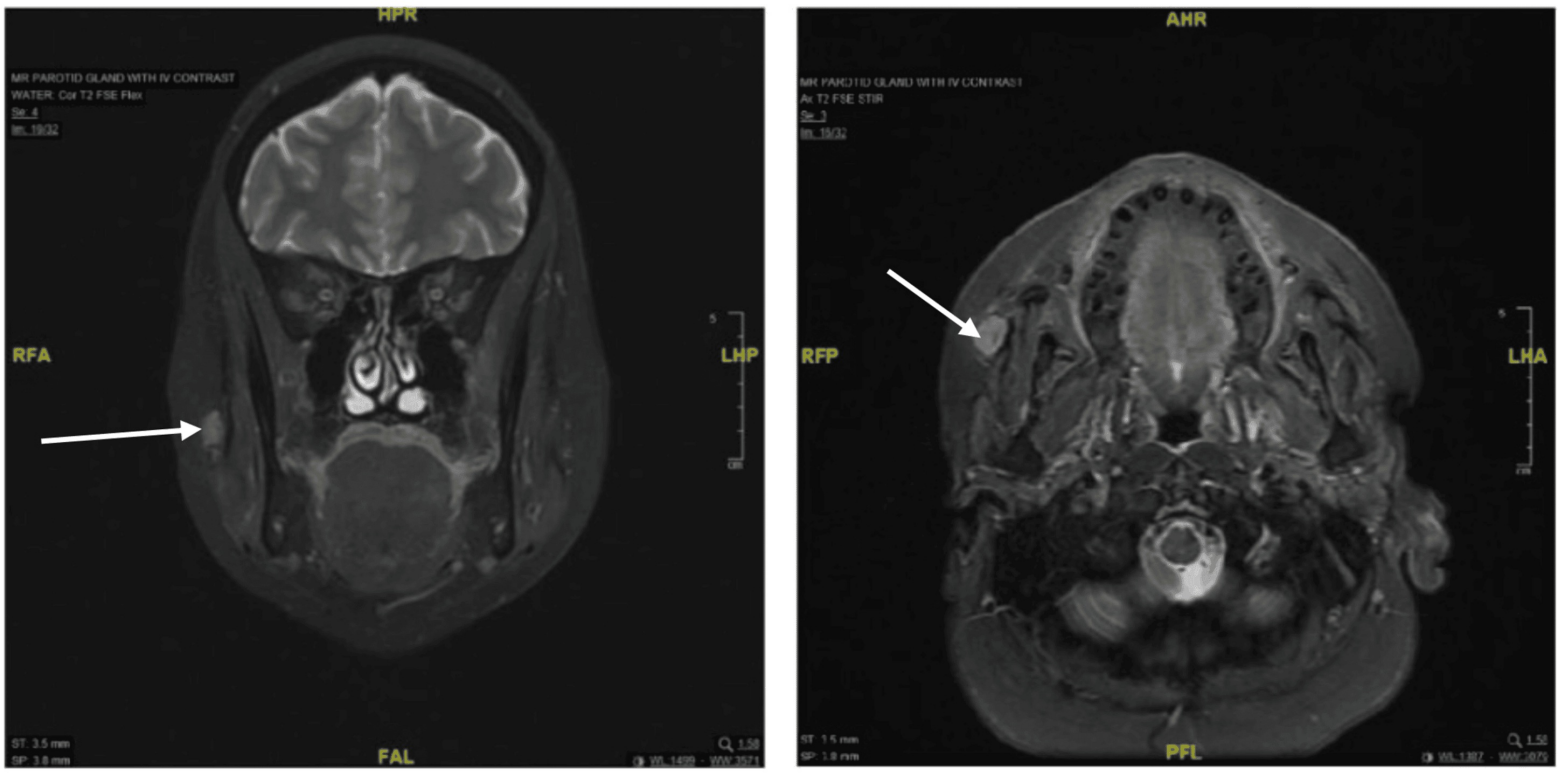
Tumor visualization in coronal and axial planes.

A 49-year-old woman presented with a progressively enlarging, non-tender right cheek swelling. Magnetic resonance imaging (MRI) of the parotid gland with IV contrast demonstrated a discrete lesion located within the deep cheek fat, extending along the right Stensen's duct, with adjacent lymphadenopathy (Figure 2). Ultrasound-guided biopsy revealed a superficial enlarged lymph node in the right cheek with cortical thickening and effaced hilum, exhibiting increased vascularity, suggestive of pathology. Bilateral cervical lymphadenopathy was noted, though deemed reactive.

Surgical resection was performed under general anesthesia. The patient was positioned supine with her head turned to the left. The right preauricular region was prepped and draped in a sterile fashion. A standard lazy-S parotid incision was made, followed by elevation of a subcutaneous flap to expose the SMAS layer. Blunt dissection allowed identification and preservation of vital anatomical structures, particularly the branches of the facial nerve. A well-encapsulated 1.5 × 1.5 cm mass located anterior to the masseter was identified and removed in its entirety via extracapsular dissection. Hemostasis was achieved using bipolar cautery, and layered closure was performed with Monocryl for deep layers and Prolene for skin. A sterile dressing and a pressure head bandage were applied. Postoperative recovery was uneventful.

**Figure 3:**
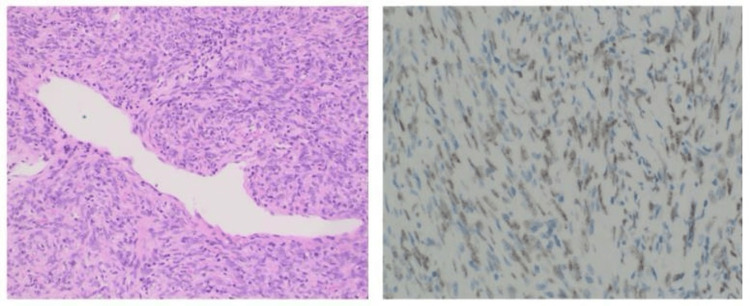
Cellular spindle cell tumor with interspersed staghorn vasculature (H&E, ×400) and a STAT6 immunohistochemical stain highlighting speckled nuclear positivity in the tumor cells.

Histopathological examination revealed a solitary fibrous tumor. Sections demonstrated a circumscribed, moderately cellular lesion composed of spindle and occasional ovoid cells arranged haphazardly or in short fascicles, with inconspicuous mitotic activity and an absence of atypia or necrosis. Ectatic, staghorn-shaped vessels and scattered lymphocytes and mast cells were noted (Figure 3). IHC analysis showed diffuse positivity for CD34, BCL-2, and STAT6 (nuclear), confirming the diagnosis. Other stains showed focal or weak positivity for CD99, Factor XIIIa, and CD10, and negative results for cytokeratin, S100, Desmin, and others. Gross tumor size was 1.5 cm; risk stratification categorized the lesion as low risk (score 0) according to the established criteria [4].

At two weeks postoperatively, the patient remained well with no complications, including no facial nerve dysfunction, sensory changes, or cosmetic asymmetry.

## Discussion

HNSFTs are rare mesenchymal neoplasms with variable clinical presentations and histologic features [5]. While SFTs can occur in all age groups, they are most commonly diagnosed in middle-aged adults [3]. Our report included two middle-aged individuals, consistent with broader demographic trends.

SFTs typically arise in the sinonasal tract, orbit, oral cavity, or neck [3]. In our cases, both tumors were located in the oral cavity and cheek. Patients typically present with a painless, slowly enlarging mass, and while pain is uncommon, it can occur in larger tumors [2].

Radiologically, SFTs are well-defined, with heterogeneous enhancement on contrast-enhanced CT and T1 isointensity/T2 hypointensity on MRI, reflecting the fibrous nature of these tumors; however, some tumors may show different signal intensities on MRI, especially if they contain areas of necrosis, myxoid degeneration, or hemorrhage, which could alter the typical T1 and T2 signals [6]. In our cases, imaging findings corresponded with the typical fibrous features.

Histologically, SFTs are characterized by bland spindle to ovoid cells in a collagenous stroma with branching vasculature, as observed in both of our cases [1]. The tumors exhibited no atypia, necrosis, or vascular/lymphatic invasion, and the mitotic rate was low. STAT6 immunohistochemical staining has been identified as a highly specific and reliable diagnostic marker for solitary fibrous tumors, distinguishing them from other spindle cell neoplasms [7]. Our immunohistochemistry findings revealed nuclear STAT6 expression, confirming the diagnosis in both cases. Other markers, including CD34 and BCL2, were also positive.

Differential diagnoses include other benign and malignant spindle cell neoplasms such as schwannomas and synovial sarcomas [8]. Careful histologic and immunophenotypic assessment, as well as radiologic correlation, are key to an accurate diagnosis [9].

HNSFTs tend to have low metastatic potential but a higher rate of local recurrence compared to other anatomical sites [9]. Although risk stratification models exist, they are less predictive in head and neck cases [10]. In our patients, both tumors were classified as low risk based on histologic and immunohistochemical features, with low mitotic activity and no necrosis. This classification aligns with a generally favorable prognosis, though long-term follow-up is advised due to the potential for recurrence [4].

Surgical excision remains the primary treatment modality for SFTs, and while margin status is debated in terms of recurrence risk, close follow-up is recommended in all cases [10]. Adjuvant radiation therapy is typically reserved for high-risk cases [1]; this was not required for our patients. Our findings emphasize the need for accurate diagnosis, individualized management, and long-term surveillance to optimize outcomes for patients with this rare tumor [1].

## Conclusions

HNSFTs are rare with variable presentations, often mimicking more common benign lesions. Our cases involving the floor of the mouth and cheek highlight the diagnostic challenges of these tumors in atypical locations. Accurate diagnosis relies on histopathological features and STAT6 immunostaining, which is crucial for confirming SFT diagnosis. Surgical excision remains the primary treatment, and both patients had uneventful recoveries with no recurrence to date. Due to the potential for recurrence or malignancy, long-term follow-up is essential. These cases emphasize the importance of considering SFTs in the differential diagnosis of head and neck masses, even in uncommon sites.
